# Progress in Bacteriology

**Published:** 1899-07-01

**Authors:** 


### PROGRESS IN BACTERIOLOGY.

**Cancer.**—Plimmer[1] reviews the views held as to the parasites of cancer from the first observations of Virchow in 1851 to the present time. He attaches the greatest importance to the work of Ruffer and Walker, whose discovery of the parasitic protozoa in 1892 aroused so much attention. The parasites may be seen in scrapings of fresh cancers. Preparations are best fixed by soaking for 24 hours in a freshly-mixed solution, platinic chloride, 15 parts; osmic acid, two per cent., four parts; glacial acetic acid, one part; they are then washed in water, in 30, 60, 90 per cent., and absolute alcohol for 24 hours, and then through cedarwood oil into paraffin. A concentrated watery solution of thionin is the best stain, and should be applied for half-an-hour. Metschnikoff classes these parasites amongst the sprotozoa, but Sanfelice and the Italian school regard them as belonging to the blastomycetes allied to the saccharomycetes. Plimmer found these organisms in 1,130 out of 1,278 cases of cancer. They are absent in degenerative patches, but marked in actively-growing cells; other forms of new growth do not contain these parasites. From a case of carcimona of the breast the author isolated an organism which, when injected into animals, caused the formation of tumours and death. The nutrient medium was an infusion of cancer with two per cent. of glucose and one per cent. of tartaric acid, and in this the parasites were grown anaërobically. On the same medium, solidified with agar, growth appears in a few weeks as a rounded yellow colony. Growth as a thick white layer takes place on potato, and also on gelatine causing liquefaction. Microscopically, the parasite is a round body with a central deep-staining portion and with a capsule. Reproduction is by budding. Inoculated intraperitoneally into guinea-pigs there results a diffused infection of new growths in all the abdominal organs, the cells of the new growth containing the specific organism. Death ensues in from 13 to 20 days. These tumours are of endothelial origin, but inoculation of the germ into the cornea of rabbits causes a true epithelial infiltration. The organisms are generally intra-cellular, they are not parts of the cell structure or any known degenerative change, and have distinct microchemical reactions.

Russell[2] summarises the work recently done in Italy in connection with the parasite of cancer. Especial attention is given to the work of Sanfelice, who has long maintained the specific pathogenic nature of the blastomycetes, and whose experimental work has been conducted with the saccharomyces neoformans. This organism, when inoculated into guinea-pigs, cats, and mice, gave rise to a fatal disease marked by proliferative tissue growth and lymphatic infection. Examination of the new growths showed them to contain bodies identical with the fuchsin bodies described in carcinomata by Russell. By inoculating blastomycetes into the peritoneum of dogs, recovering them from the lymph glands, and passing them through other dogs, Sanfelice has succeeded in producing epithelial growths (adeno-carcinomata) with metastases. Roncali investigated several carcinomatous growths, and in each demonstrated the parasite as both extra and intracellular. From one case he obtained cultures of blastomycetes, which, when inoculated into guinea-pigs, caused death with the production of granulomatous tumours. In these tumours there were parasites identical with a blastomyces isolated by him from an epithelioma of the tongue. In the same way Busse, Kahane, Corselli, and Frisco have all obtained cultures of blastomycetes from malignant tumours, and these parasites when inoculated into guinea-pigs, rabbits, and dogs produced similar growths.

Foulerton[3] states that until recently the only blastomycetes regarded as pathogenic to man were the S. albicans (vulg. oidium alb.), S. subcutaneus tumefaciens, described by Curtis, and B. dermatitidis (Gilchrist). Sanfelice's results he regards as uncorroborated. Many varieties of yeast are pathogenic to animals. Thus, of ten yeasts tested by Foulerton, nine were pathogenic and seven gave rise to tumours at the site of inoculation or to secondary nodular growths in internal organs. From the new growths cultures of the original yeast were made. A new variety of saccharomyces named the tumefaciens albus was found most actively pathogenic for tame mice and rabbits, with the production of numerous new growths which are histologically granulomata at the site of inoculation, and in the deeper tissues masses of round cells, mononuclear, with homogeneous fibrinoid ground tissue. Within these cells the yeast organism could be seen. The evidence brought forward by Sanfelice and Roncali is so scanty that we are not justified in saying more than that some so-called sarcomata are really cases of yeast infection. As to the exact causation of carcinoma we are still absolutely ignorant.

**B. aerogenes capsulatus. Air Embolism.**—Since the first description by Welch and Nuttall, in 1892, many cases of infection by the bacillus aërogenes capsulatus have been reported. A summary of these published before 1896 is to be found in the *Journal of Experimental Medicine*, 1896. Norris[4] reports six cases of general infection. The B. capsulatus aërogenes is from 2-8 $\mu$ in length, and is often found as a diplo-bacillus. It stains with Gram, and the capsule may be demonstrated by treating with glacial acetic acid, and then staining with an aqueous solution of gentian violet. The organism is anærboic. On glucose agar, broth, potato, milk, &c., growth is abundant, and generally accompanied by much gas formation. In agar plates the colonies are ovate, opaque, granular at the edge, and of a purplish tint. In life the bacillus has commonly been associated with emphysematous cellulitis, and its presence in the tissues after death gives rise to the condition known as "foam-organs," and a general gaseous infiltration of the parenchymatous tissues. Norris's cases were as follows—(1) Rupture of cervix uteri, followed by general infection; (2) acute leucæmia, with terminal infection; (3) hypertrophic cirrhosis of liver; (4) gaseous phlegmon, following compound fracture; and (5) hæmorrhagic pancreatitis. Howard[5] describes a case of fibrino-purulent cerebrospinal meningitis, ependymitis, abscesses of the cerebrum, gas cysts of the cerebrum, cerebrospinal exudation, and of the liver due to the same bacillus. Herring and Renling[6] also describe a case in which cavities were produced in the brain by the B. aërogenes capsulatus, the initial lesion and probable seat of inoculation being a perforating gun-shot wound of the abdomen. There can be no doubt that the majority of cases of gas in the tissues, either during life or at post-mortem examination, and which formerly were regarded as cases of "air-embolism," are due to infection by this organism.

**Simple Posterior Basic Meningitis of Infants.**—Steel[7] claims that this is a specific disease due to a particular micro-organism. This organism he found in seven out of eight consecutive cases, examined post-mortem. It is distributed throughout the exudation and in the cerebrospinal fluid. It is a diplococcus with the opposed surfaces flattened. The diplococci may be found free or within the cells. On nutrient media the tendency is rather to grouping in pairs than to forming chains. There is no capsule. All the ordinary stains are efficient, but the organism is decolourised by Gram. It is aërobic, and grows well on agar at 37 deg. C., forming small greyish-white viscid colonies. On blood agar growth is more rapid and the colonies bigger and more opaque. There is abundant growth with formation of fine sediment in broth, and milk is not coagulated. As the organisms are scanty in the exudation, it is suggested that a piece as large as a pea be taken and spread over the medium. Not less than six inoculations should be taken. The exudation is thickened lymph, not pus, and in some cases is also found in thickened tissues round the joints. The diplococcus is pathogenic to guinea-pigs, mice, and rabbits. The vitality of this organism is much greater than that of the pneumococcus or D. intracellularis, though it is possible that it may be a modification of the last.

[1] The Practitioner, April, 1899. [2] Lancet, April 29, 1899. [3] Jour. of Path. and Bact., May 1899. [4] Amer. Jour. of Med. Sciences, Feb., 1899. [5] [6] Bulletin of the Johns Hopkins Hosp., April, 1899. [7] Jour. of Path. and Bact., May, 1898.

